# Supplementary material for: Are carbon emissions trading and green financial instruments synergistic? -Comprehensive quantitative research based on content analysis
Source: PLoS One. 2024 Mar 7;19(3):e0298601. doi: 10.1371/journal.pone.0298601 (PMC10919601; doi:10.1371/journal.pone.0298601)
Supplement: S1 Data — (ZIP) [file pone.0298601.s001.zip › Data/地级市Z_F工作报告.zip!/║■─╧/╘└╤⌠╩╨/└┤╫╘╣1⁄2╓┌║┼í╛┬φ┐╦╩2╛▌═°í┐_2016.docx]

各位代表：

现在，我代表市人民政府，向大会报告政府工作，请予审议，并请市政协委员和其他列席人员提出意见。

“十二五”及2015年工作回顾

“十二五”时期是岳阳发展历程中极不平凡的五年，也是我市改革发展取得重大成就的五年。在中共岳阳市委坚强领导下，我们深入贯彻党中央、国务院和省委、省政府各项决策部署，团结拼搏、克难奋进，较好完成“十二五”规划任务，成功获评全国文明城市、全国绿化模范城市、全国宜居城市百强、中国最具幸福感城市、中国最具文化软实力城市等荣誉称号，岳阳正以新的面貌、新的姿态、新的形象，阔步迈上“一极三宜”江湖名城建设新征程！

经济实力实现新跨越。2015年全市GDP达2886.3亿元，比2010年基本翻一番，年均增长10.9%。规模工业增加值年均增长12.8%，固定资产投资、社会消费品零售总额、公共财政预算收入、城乡居民人均可支配收入分别达2154.7亿元、1020.6亿元、302.8亿元、25202元和 12091元，年均分别增长27.7%、14.4%、16.7%、10.8%和13.9%。县域经济实力增强，经开区公共财政预算收入突破30亿元，6个县市区过10亿元，汨罗进入全省县域经济十强。

转型升级取得新成效。三次产业结构继续优化，由“十一五”末14∶54.2∶31.8调整为11∶50.1∶38.9。形成石化、食品两大千亿产业和10个省级以上工业园区，湖南岳阳绿色化工产业园获批国家首批低碳工业试点园区，岳阳电子商务产业园获批国家电子商务示范基地。2015年全市园区工业增加值占规模工业比重60.1%，非公经济占GDP比重62.1%，高新技术产业占GDP比重18.6%，较2010年提高6.1个百分点。实现旅游总收入332.7亿元，年均增长26.5%。市级以上农业产业化龙头企业267家，农业综合机械化水平67%，完成环洞庭湖基本农田建设重大工程，屈原、华容跻身国家现代农业示范区，君山获评全国农产品质量安全示范区，岳阳县获评全国绿色食品原料标准化生产基地。万元GDP能耗年均下降6.6%，主要污染物排放削减率超额完成省定任务，岳阳获评全国节水型社会建设示范区。

口岸经济获得新突破。综合保税区、汽车整车进口口岸、进口肉类指定口岸、进口粮食指定口岸、固废进口指定口岸获批运营，城陵矶港纳入启运港退税政策试点范围，成为全国唯一拥有6个国家级开放平台的地级市。成功开通城陵矶至港澳台和上海洋山港直达航线、21世纪海上丝绸之路岳阳—东盟接力航线。湖南城陵矶国际港务集团正式运营，岳阳港获批全国绿色循环低碳港口试点，集装箱吞吐量、进出口总额年均分别增长13.9%、24.1%。岳阳开放开发风帆正举，其势已成，来势更加看好，走势更加强劲！

城乡面貌得到新改观。开展“五创提质”、“秀美系列”评选和“美好社区、温馨家园”创建活动，中心城区严重低洼渍水地段整治成效明显，改造小街巷1153条，市体育中心、王家河流域生态休闲和历史文化记忆长廊等标志性工程加快建设，污水日集中处理能力比“十一五”末提高6.5倍，城镇垃圾无害化处理率98%，城镇化率54%。城乡基础设施大为改善，蒙华铁路岳阳段加快推进，随岳、通平、岳常、石华高速和芙蓉大道湘阴段建成通车，公路通车里程突破2万公里；实施农田水利“百千万”工程，完成中小河流治理、水库除险、堤防加固等各类工程22.1万处；投产输变电项目59个，升级改造农网2445个村。完成湘江保护与治理第一个“三年行动计划”，开展“洞庭风雷”行动，加强铁山水资源保护，是全省唯一设立重要水源地保护专项资金的市州。

深化改革迈出新步伐。启动实施9大类109项改革，发展活力与动能更加强劲。深化农村改革。率先全省完成国有林场改革；农村土地承包经营权确权登记颁证、农民住房财产权抵押贷款试点扎实推进；实施农业“三项补贴”改革；临湘列为全省第一批粮食适度规模经营“财银保”项目试点县，华容列为全省唯一农业改革试点县。深化经济体制改革。投融资体制、商事登记制度改革有效推进；引进3家股份制商业银行、2家村镇银行，新增3家上市企业；建立全口径政府预算管理体系，预决算和“三公”经费全面公开，“营改增”试点全面落实；基本完成第一轮国有企业改革。深化行政体制改革。完成市本级机构改革任务，压减各类行政许可事项160项，精简46.1%，率先全省不再保留非行政许可事项；实施乡镇区划调整改革，撤并乡镇52个，调减1/3；优化调整中心城区管理体制，组建南湖新区、城陵矶新港区；临湘列为全国“多规合一”试点市，平江列为省直管县经济体制改革试点县。

社会事业开创新局面。五年建成义务教育合格学校、农村公办幼儿园830所，创建国家改革示范职业学校5所，职业教育、未成年人思想道德建设获评全国先进，湖南理工学院获批硕士学位授予单位。广泛开展爱国主义教育和精神文明创建活动，巴陵戏《远在江湖》获田汉大奖。成功举办全国体育舞蹈大赛、全省第八届少数民族运动会。完成县级医院、乡镇卫生院改扩建等项目599个，人口年均自然增长率6.5‰。新增各类保障房7.5万套，完成棚户区改造15.5万户、农村危（旧）房改造7.7万户。城乡居民养老保险、新农合、城镇医保覆盖率97%以上。城乡低保、五保保障标准进一步提高，特困家庭大病医疗救助获第八届“中华慈善奖”。新增城镇就业27.8万人、农村劳动力转移就业31.9万人。解决农村饮水不安全人数278.4万人。实施公交优先“三年行动计划”，更换新能源公交车415台。完成第九次村委会换届选举和“六五”普法任务。安全生产、信访工作保持全省先进，岳阳楼区获评全国社区治理和服务创新实验区。

2015年是“十二五”收官之年。面对复杂多变的宏观环境、持续加大的经济下行压力、艰巨繁重的改革发展稳定任务，我们全力统筹稳增长、调结构、促改革、惠民生、防风险，经济社会持稳发展。全市GDP增长8.7%，规模工业增加值增长8.3%，固定资产投资增长20.4%，社会消费品零售总额增长12.7%，公共财政预算收入增长18.1%，城乡居民人均可支配收入分别增长9%、9.3%，主要经济指标增幅居全省第一方阵。一年来，我们突出抓了以下工作：

一是抓重大项目建设。牢固树立“项目为大、项目为重、项目为要”理念，组织举办4次“一区一港四口岸”专题招商推介会、4次重大项目集中开工和集中竣工仪式，形成大干快上项目建设热潮。持续开展禁拆治违、土地依法征收“百日攻坚”行动，中心城区拆除违法建设62.9万平方米，依法征收土地10761亩，是近年工作力度最大的一年。“双百工程”重大项目开工115个、竣工69个；岳阳三荷机场、洞庭湖博览园、神华国华岳阳电厂、华电平江电厂等一批打基础、管长远的项目开工建设；高速公路“两路一桥”、临湖公路、环南湖旅游交通三圈等一批重点督办的项目加快推进；综合保税区一期、京港澳高速岳阳收费站改扩建等一批重大项目投入运营；株冶绿色改造升级、中粮城陵矶产业园等一批项目成功落地；岳阳端午文化产业整体开发、云溪静脉产业园生活垃圾焚烧发电等9个项目进入全国、全省PPP示范项目库。

二是抓新增长点培育。一年来，我们充分发掘经济增长新动力，以新增长点支撑新增长极建设。大力培育园区经济增长点。市委、市政府出台《关于进一步促进园区产业发展的意见》，强力推进“135”工程，竣工标准化厂房204.5万平方米，超年度计划21%。出台支持大众创业、万众创新政策，新登记企业主体6688家，增长24.3%。经开区获批国家级小微型企业创新创业示范基地。大力培育旅游经济增长点。围绕旅游总人数、总收入两年翻番目标，谋划推进君山岛景区（芦苇荡）和野生荷花世界景区整体开发，创新岳阳楼景区旅游收入激励机制，成功举办“天下江湖、畅游岳阳”、第二届黄茶文化节、第八届洞庭湖国际观鸟节等活动，牵手中央电视台《乡约》栏目走进岳阳。石牛寨、亚马逊水上乐园等景区人气火爆，平江旅游异军突起。尽管目前我市旅游经济还是经济发展中的一块短板，但只要长抓不懈、久久为功，旅游强市梦想是一定可以实现的！大力培育新兴业态增长点。以新兴业态抢占发展制高点，全力扶持电子信息、生物医药、节能环保等产业发展，新兴产业增加值占规模工业比重22.2%。岳阳获批“宽带中国”示范城市，汨罗列为全国电子商务进农村综合示范县。大力培育县域经济增长点。开展“学长沙、赶宜昌、超九江”活动，出台“三年行动方案”，13个县市区实行“一对一”赶超；创新县域经济评价办法，明确“六个一”工作重点，发展导向更加明确、路径更加清晰、抓手更加集中。

三是抓民生福祉改善。加大财政民生投入力度，2015年完成25项38件省市民生实事。率先在全省启动规范农村村民建房试点，强力推进城市棚户（旧城）区和农村危（旧）房改造，中心城区“十大片区”改造全面铺开，1.97万农户享受到农村危房改造惠民政策，是近年来规模最大、成效显著的一年，“两房两棚、两供两治”工作经验在全省推介。启动“精准扶贫三年攻坚行动”，着力帮扶412个贫困村，脱贫8.1万人。出台高校毕业生就业创业扶持政策，大学毕业生就业动态清零。开展大气污染防治攻坚行动，空气质量达标率88.3%。成功应对中心城区“4·03”特大暴雨、临湘“6·01”特大山洪地质灾害、“东方之星”客轮翻沉事件，防汛抗旱救灾有力有效。

四是抓社会治理创新。以改革精神推动社会治理创新，把政府建到群众家门口。推进政务服务重心下移，支持岳阳楼区将85项公共服务事项下放社区办理，打通服务群众“最后一公里”。启动社区网格化管理试点，探索推进居民自治，社会治理服务能力进一步增强。推行大城管模式和火车站地区综合执法，持续开展“打非治违”、“治理车辆超限超载”、“五打五整治”等专项行动，推进“信访工作双向规范”主题活动，安全生产形势持稳向好，综治民调排名全省第二位，屈原、君山分别排名全省第三、第五位。

其他各项事业协调发展，国防动员和后备力量建设取得新成绩，武警、消防、国安、人防、统计、审计、史志、档案、保密、老龄、民宗、科协、残联、气象、外侨、联络接待、公积金、移民、烟草、盐业、邮政、通信等工作实现新进步，工会、共青团、妇联及各民主党派、工商联、无党派人士在经济社会发展中作出新贡献。

各位代表，5年的发展成就有目共睹，5年的工作经验弥足珍贵。这就是：必须坚持科学谋划。遵循发展规律，理清发展思路，突出发展重点，坚持一张蓝图干到底，一任接着一任干，保持定力，精准发力，确保了岳阳好的发展势头。必须坚持改革创新。主动适应引领经济发展新常态，破除思维定势、工作惯性和路径依赖，以思想观念创新、体制机制创新、工作方法创新开创发展新局面。必须坚持民生为本。把保障改善民生作为政府工作的出发点和落脚点，持之以恒办实事、抓脱贫、兜底线、解难题，让广大群众共享更多改革发展成果。必须坚持依法行政。将法治思维、法治方式贯穿政府工作全过程，依法履行政府职责，认真办理市人大议案、代表建议和市政协建议案、委员提案，加快建设法治政府。必须坚持从严从实。严格执行中央“八项规定”，深入开展党的群众路线教育实践活动、“三严三实”专题教育和“三提升”主题活动，认真落实市委“三个办法”，努力提振广大干部的精气神，广泛凝聚干事创业的正能量！

各位代表，岳阳经济社会发展取得的重大成就，是省委、省政府和市委正确领导的结果，是市人大、市政协监督支持的结果，是全市上下共同努力的结果。在此，我代表市人民政府，向全市人民，向所有关心支持岳阳改革发展的社会各界人士，表示衷心的感谢和崇高的敬意！

成绩令人鼓舞，发展任重道远。我们清醒认识到，岳阳发展还面临不少困难和问题。主要是：经济增长动能不足，新增长点培育还有待加快；经济结构不优，县域经济、开放型经济、金融经济还有待突破；民生改善任务繁重，社会保障能力还有待提高；风险隐患依然较多，社会治理创新还有待加力；行政效能仍需提升，政府自身建设还有待加强。对这些困难和问题，我们将采取有力措施，认真加以解决。

“十三五”发展目标和主要任务

“十三五”时期，是全面深化改革的攻坚期、全面建成小康社会的收官期、全面推进依法治市的关键期，也是全球经济进入大调整大转型、国内经济步入中高速发展新常态的挑战期，更是岳阳推进“一极三宜”江湖名城建设的战略机遇期。未来五年，我国经济长期向好的基本面没有变，经济韧性好、潜力足、回旋空间大的基本特质没有变，经济持续增长的良好支撑基础和条件没有变，经济结构调整优化的前进态势没有变，特别是国家“一带一路”、洞庭湖生态经济区、长江经济带、长江中游城市群、“两横三纵”城市化，以及湖南“一核三极四带多点”区域布局等一批重大战略深入实施，岳阳面临的战略机遇持续叠加，必将进入新一轮发展“黄金期”。我们必须坚定信心、抢抓机遇、加速赶超，在引领发展新常态中赢得主动，在集聚发展新动能上取得突破，全力推动经济“双中高”发展，全力推动岳阳迈向基本现代化。“十三五”时期政府工作总体要求是：认真贯彻落实党的十八届五中全会、省委十届十五次全会、市委六届十二次全会精神，按照“五位一体”总体布局和“四个全面”战略布局，贯彻创新、协调、绿色、开放、共享发展理念，促进“三量齐升”，实施“五化同步”，推进“621”行动，加快转型升级，深化改革开放，强化生态保护，统筹城乡发展，保障改善民生，确保全面建成小康社会，加快建设“一极三宜”江湖名城。

主要目标是：

——确保“一个高于”：主要经济指标增速高于全省平均水平，GDP迈上5000亿元台阶，公共财政预算收入迈上500亿元台阶。

——实现“两个率先”：率先在洞庭湖地区全面建成小康社会，率先在全省建成通江达海、产业兴旺、开放共赢、绿色发展的新增长极。

——达到“三大提升”：经济总量占全省比重有较大提升，达到12%以上；发展质量和效益有较大提升，高新技术产业增加值占GDP比重24%以上，万元GDP能耗下降15%；人均均量和群众获得感有较大提升，城乡居民人均可支配收入突破3万元，全民素质和社会文明程度明显提高。

主要任务是：

一、坚持跨越发展，全面加快新增长极建设

今后五年，我们要扩大有效供给与需求，既要稳住速度，又要提高效益，确保经济实现更高水平、更高质量、更可持续发展。

突出新型工业带动。岳阳工业有基础、有潜力、有希望，要强化“地方实力看工业”的理念，充分发挥工业主导经济增长作用，力争规模工业增加值年均增长10%左右。做大做强工业园区，进一步完善基础设施，创新体制机制，大力招商引资，“十三五”期间力争4个园区技工贸总收入突破千亿元，6个园区突破500亿元，园区工业增加值占规模工业比重70%以上。培育发展新兴产业，积极对接“中国制造2025”和“互联网+”，实施重大产业和重点企业帮扶行动，支持现代装备制造、军民融合、生物医药、电力能源、节能环保等产业发展，培育壮大优势企业、骨干企业，引领工业向中高端水平迈进。

突出有效投资拉动。当前，岳阳投资总量不是多了而是少了，投资效益不是高了而是低了，必须充分发挥有效投资拉动经济增长的关键作用，力争固定资产投资年均增长18%。积极对接国家、省“十三五”发展重大战略，紧跟政策走向、资金投向，加强项目开发储备，深挖基础设施、生态环保、扶贫开发、民生保障等领域投资潜力。维持合理的政府投资力度，用好专项建设基金、地方融资平台、债券市场等工具，发挥政府投资“四两拨千斤”的作用。拓宽非公企业、民间投资领域和空间，破解准入限制、项目审批等制度性障碍，在更大范围、更广领域激发社会资本参与投资的热情。

突出消费需求推动。顺应多样化、个性化消费需求趋势，培育新的消费热点，发展新的消费模式，力争社会消费品零售总额年均增长13%。重点促进移动互联网、健康养老、旅游、信息、绿色等新兴消费，完善云计算、大数据平台，提升金融、物流、零售等领域在线化、智能化水平。着力化解房地产库存，推动房地产业持稳健康发展。把旅游作为第三产业龙头来抓，加快构建以岳阳楼景区、岳州古城等为重点的古城旅游板块，以洞庭湖博览园、环南湖“13118”综合工程等为重点的江湖旅游板块，以天岳幕阜山、大云山等为重点的山岳旅游板块，打响“游山玩水登楼逛古城”旅游新品牌。谋划推进游客服务中心、主题公园、旅游公路等配套建设，促进旅游与文化深度融合，加快建设令人流连忘返的旅游目的地，把旅游业培育成投资的重点、消费的热点、开放的亮点！

二、坚持开放发展，不断提高外向型经济水平

今后五年，我们要依托岳阳拥有163公里长江岸线资源、占有洞庭湖50%以上水域面积、作为湖南唯一通江达海港口城市的优势，全方位拓展外向型经济发展新空间，开创开放发展新局面。

筑牢港口平台。我市拥有“一区一港四口岸”开放平台优势，一定要利用好这个优势，发挥好这个优势。要在“一区一港四口岸”的基础上，建成运营湖南航运交易所、汨罗公路口岸、七里山铁路口岸和岳阳电子口岸，构建现代口岸体系。整合港口水运资源，加快港口码头群建设，实施荆江航道整治二期工程，打造长江中游区域性航运物流中心。用好用活启运港退税政策，推进21世纪海上丝绸之路岳阳—东盟、岳阳—澳大利亚接力航线和城陵矶至港澳台直航常态化，开通城陵矶至日本、韩国、东南亚等近海直达航线，拓宽湖南通往世界的出海口。

培育开放主体。依托岳阳三荷机场区位和空域优势，着眼中国大陆货物航空核心枢纽城市和低成本客运航空基地的战略定位，加快规划建设空港产业园。深化与上港集团合作，做大做强湖南城陵矶国际港务集团。加快城陵矶综合物流园、临港冷链和农产品物流园、华菱欣港物流总部经济、南翔万商国际商贸物流城等项目建设。扶持5—10家年营业额10—20亿元物流企业，引进2—3家10亿美元以上加工贸易企业，新增10家上市企业，吸引更多开放主体集聚岳阳，推动开放经济扩量倍增。

加强区域合作。积极对接国家“一带一路”和湖南“一带一部”战略，支持本土企业到海外扩张市场，鼓励外地客商来岳投资创业。加强与沿江环湖和高铁沿线城市在基础设施、航运物流、旅游开发等领域合作，促进区域融合发展。深化与长株潭城市群合作，加快“两型社会”滨湖示范区建设，大力发展“飞地经济”，支持长沙经开区汨罗产业园发展，打造区域共建共享示范窗口。

三、坚持创新发展，充分激发市场活力

今后五年，我们要加快实施创新驱动发展战略，确保在重要领域、关键环节取得突破，坚决破除发展的体制机制障碍。

深化改革创新。科学把握改革力度与节奏，突出抓好供给侧结构性改革。深化行政体制改革，继续精简行政审批事项。统筹化解国企改制遗留问题，加快建立健全现代企业制度。推进财政体制改革，强化全口径预算管理，实行跨年度预算平衡机制和中期财政规划管理，完善政府举债融资机制。深化金融体制改革，规范民间投融资市场和互联网金融，发展普惠金融，提高金融服务实体经济效率。统筹抓好农村土地征收、集体经营性建设用地入市、宅基地制度改革为代表的“三块地”改革和社会领域改革，激发市场活力和社会创造力。

加快科技创新。发挥科技创新引领作用，依托骨干企业、高等院校、科研机构新建一批重点实验室、企业技术中心和科技成果转化中心，“十三五”期间新增17家省级以上技术创新平台、5支省级科技创新人才团队，推进产学研用深度融合。强化企业创新主体地位和主导作用，鼓励企业开展产品创新和技术革新，新增发明专利授权量800件、高新技术企业70家。健全科技成果转化利益共享机制，增强企业和园区科技创新孵化能力。

支持创业创新。实施“百优资助工程”，加强创新创业基地建设，积极发展众创、众包、众扶、众筹空间，新增创业基地13个、众创空间10个、创业主体15万个以上。鼓励有意愿、有实力、有技能的创业者就地创业、返乡创业，形成大众创业、万众创新“千帆竞发”的民营经济发展态势。

四、坚持协调发展，大力推进城乡一体化

今后五年，我们要科学统筹城乡发展，推动城乡要素平等交换和公共资源均衡配置，形成以城带乡、城乡互动的一体化格局。

推进新型城镇化。坚持规划先行，强化规划管控，推动“多规合一”，实现县城、乡镇和中心村规划全覆盖。拓展城市发展空间，加快南湖新区、洞庭新城、高铁新城、空港片区、芭蕉湖片区等重点区域开发，加强地下综合管廊、海绵城市、人防工程和数字城管、智慧交通建设，积极推广大城管模式，增强城市综合承载功能。推动县城和重点镇协调发展，着力打造一批具有示范作用的沿江中心镇、省际边界镇、特色产业镇、历史文化名镇。加快农民工市民化，稳步提高户籍人口城镇化率，力争城镇化率达到60%。

推进农业现代化。加快农村一、二、三产业融合发展，推动资源、要素、技术、市场需求在农村整合集成和优化重组。大力发展粮食、水产、茶叶、蔬菜、饲料等优势产业，重点打造“一个300万亩、三个50万亩”特色生产基地。加快岳阳台湾农民创业园、洞庭湖绿色食品产业园和屈原、华容国家现代农业示范区建设。重点扶持20家农业产业化龙头企业、100个特色农业产业园区，大力培育家庭农场、农民合作社等新型农业经营主体。引导工商资本下乡，促进农村土地有序流转，鼓励农业适度规模经营，推广良种良法和先进适用农机，加快农村电商发展。

统筹推进城乡基础设施建设。科学整合项目资金，形成集中财力办大事的投入机制，构建完善城乡交通、水利、能源、信息四张网。统筹城乡交通建设。建成运营岳阳三荷机场、蒙华铁路岳阳段、平益高速公路；启动岳长、长平汨城际铁路，常岳九、月益铁路岳阳段建设，谋划推进环洞庭湖公路、湘江北路建设，构建水陆空综合交通体系。统筹城乡水利建设。完成洞庭湖区沟渠疏浚任务；大力实施中小河流治理、城镇防洪和三峡后续影响工程；加快推进小农水项目县、农田水利“百千万”工程，实现100%乡镇、90%行政村通自来水。统筹城乡能源建设。加快小墨山核电、福寿山抽水蓄能电站等项目进程；完成农村电网升级改造；基本实现重点乡镇燃气管道化。统筹城乡信息化建设。实施城市基础网络完善工程和“宽带乡村”工程，推进城区光纤到户和“三网融合”，建设“无线”城市。通过统筹城乡基础设施建设，让城市享有农村的清新秀美，让农村享有城市的现代文明！

五、坚持绿色发展，加快建设水墨丹青岳阳

今后五年，我们要把生态文明建设融入经济社会发展全过程，强化生态环境保护和治理，让绿水青山与金山银山共建共生。

严格推进节能减排。实施节能减排低碳发展行动计划，推动华能电厂、巴陵石化、长岭炼化等重点企业节能改造，确保万元规模工业增加值能耗降低20%；加快淘汰落后产能，严禁新上“三高一资”项目，关停一批环境污染大、资源消耗高企业。实施循环发展引领计划，推进湖南岳阳绿色化工产业园循环化改造试点、汨罗循环经济和临湘资源型城市转型发展。推广建筑节能技术和可再生能源建筑应用，加快形成绿色生态、绿色生产、绿色消费“两型”发展模式。

严格整治生态环境。实施大气污染防治行动，推进电力、石化等行业脱硫脱硝工程，加快淘汰“黄标车”，让“岳阳蓝”成为城市空气质量的一种品牌。加强水环境整治，落实最严格的水资源管理制度，强力推进洞庭湖水环境综合整治、湘江保护与治理省政府“一号重点工程”，加强中心城区“四湖两河”和汨罗江、新墙河、华容河、黄盖湖、松杨湖等河湖治理。对破坏生态环境的人和事，坚决做到“零容忍”、出重拳、严处罚，不管涉及到谁，都严格追究、决不姑息，只有这样才能留住岳阳的蓝天白云、绿水青山！

严格保护生态功能区。坚持保护优先、自然恢复为主，加强洞庭湖湿地保护，提高生态涵养功能。加强国家、省级森林公园保护与开发，大力植树造林，提高生态承载能力。加强水源地保护和重要水源地达标建设，突出抓好铁山水库外引水源工程和“两保”工作，规划建设金凤湖生态示范区，力争重要水功能区、饮用水源地水质达标率分别提高到95%和100%，让广大市民始终喝上安全水、放心水、优质水！

六、坚持共享发展，努力提升人民幸福指数

今后五年，我们要把保障改善民生摆在重中之重位置，让全市人民的日子过得更加美好、更加红火。

推进精准扶贫。全面建成小康社会，最艰巨、最繁重的任务在贫困地区，党和政府最牵挂、最关切的焦点在贫困地区。要强力推进“精准扶贫三年攻坚行动”，重点以平江山区、铁山库区作为扶贫攻坚主战场，加快实施基础设施“六到农家”工程和公共服务“六个落实”，确保贫困村电网改造、交通通畅、互联网覆盖、饮水安全比率达100%。强化产业扶持、转移就业、易地搬迁、教育支持、医疗救助、社保政策兜底等措施，确保35.2万贫困人口整体脱贫，坚决打赢脱贫攻坚战，让贫困群众与全市人民共建全面小康、共享美好生活！

繁荣社会事业。坚持教育优先。均衡配置教育资源，“十三五”期间全市新增初级中学、城镇小学、公办幼儿园123所，加快农村偏远地区寄宿制学校和教师队伍建设，建成20个省级特色专业和教育公共信息云服务体系，加快普及高中阶段教育，支持湖南理工学院创办全省一流高校。建设健康岳阳。广泛开展全民健身运动，加快市体育中心、全民健身馆建设；推进县级公立医院改革，完成县级医院和乡镇卫生院标准化建设，新建城市医养服务中心10个，实现居民健康“一卡通”；坚持计划生育基本国策，全面落实一对夫妇可以生育两个小孩政策。发展文化事业。加快市档案馆、图书馆、科技馆、青少年活动中心、妇女儿童活动中心等文化基础设施建设，新建一批社区和村级文化中心，实施文化惠民工程，加大精品创作力度，丰富群众文化生活。

健全保障体系。建立完善城乡统一的公共就业服务体系，推进屈原统筹城乡就业示范区建设，实施“零就业”家庭、大学毕业生就业动态清零，城镇登记失业率控制在4.3%以内。稳妥推进机关事业单位和企业职工养老金并轨，实行基本医疗保险异地就医结算，加大医疗救助和贫困家庭重特大疾病救助力度，稳步提高城乡低保标准。推进住房保障货币化，完成中心城区“十大片区”棚户（旧城）区改造和农村危（旧）房改造三年计划，逐步规范农村村民建房，着力解决“安居难”问题，与广大困难群众一道协力共建“暖心房”、早日共圆“安居梦”！

加强社会治理。推进法治岳阳、平安岳阳建设。建成社会治安立体防控体系，深入开展禁毒人民战争，依法打击各类违法犯罪。突出抓好安全生产和车辆超限超载整治，严厉“打非治违”，坚决遏制重特大事故发生。实施质量强市战略，建成食品安全检测中心，强化农产品、食品药品质量安全监管，严惩制售假冒伪劣行为。加大非法集资打击力度。推广社区网格化管理，实现政府治理和社会调节、居民自治良性互动。推进阳光信访、责任信访、法治信访。健全社会诚信体系，积极开展志愿者服务，巩固提升文明创建成果。

2016年工作重点和主要措施

2016年是“十三五”开局之年，做好今年工作至关重要。必须全面落实中央、省委、市委经济工作会议精神，在适度扩大总需求的同时，坚定不移去产能、去库存、去杠杆、降成本、补短板，全力推进经济社会平稳健康发展。主要发展目标是：GDP增长9%左右，规模工业增加值增长9%左右，固定资产投资增长18%，社会消费品零售总额增长13%，公共财政预算收入增长10%左右，城乡居民人均可支配收入分别增长9.5%和10%。实现上述目标，要突出抓好五项工作：

一要加快实施一批重大项目。坚持把“大抓项目、抓大项目”作为一种常态，深入开展项目大招商、大竞赛、大帮扶活动，推动项目建设攻坚提速。以重大产业项目推动经济转型发展。重点抓好军民融合产业园、中粮城陵矶产业园、神华国华岳阳电厂、华电平江电厂、中航浮空器、株冶绿色改造升级等项目建设，支持长岭炼化、巴陵石化等技改升级，鼓励中航长动、金秋红日等企业“退二进三”。推进韩国首尔大学岳阳数字化医院、友谊阿波罗、金悦洋商业广场、海吉星农产品市场等项目建设。以基础设施项目提升综合承载能力。推进杭瑞高速洞庭湖大桥、岳阳东和岳阳南客运枢纽站、皇姑塘立交桥、武警长沙直升机场进场公路和G240岳阳城区至湘阴段、临湖公路城区段、进港铁路、松杨湖鸭栏专线等项目建设，建成岳望、大岳高速公路和京港澳高速新开联络线。启动第二批农村水电增效扩容改造。投产4个110KV、5个35KV输变电项目。推进钱粮湖垸、大通湖东垸蓄洪安全工程建设，完成集成安合、白泥湖、义合金鸡、君山垸加固工程扫尾和23座重点病险水库整治、474座水库除险加固、65平方公里水土流失治理。以生态环保项目构筑绿色保护屏障。扎实推进洞庭湖水环境综合整治、湘江保护与治理第二个“三年行动计划”，继续开展“洞庭风雷”、南湖水环境综合治理、铁山水库等饮用水源地保护攻坚行动，加快推进整县市区农村环境整治，坚决守住生态保护红线。

二要强力推进一批升级行动。岳阳发展已经到了一个十分重要的节点，必须牢牢抓住发展的“牛鼻子”，强力推进“五区”建设，构建多元支撑、多点发力的新格局，打造岳阳发展升级版。进一步提升城区品位品质。加快城市总体规划修改。抓好中心城区“十大片区”棚户（旧城）区改造和南湖、芭蕉湖区域开发建设，将“十片两湖”区域逐步建设成为中心城区宜居宜业宜游的新亮点。推进九华山、珍珠山公园建设和城市主干道、桥梁立体绿化改造，丰富城市空间景观。完善城区道路微循环系统，加快治理交通“堵点”，规划建设一批停车场。进一步提升园区发展水平。全面落实市委、市政府“园区十条”，对省级以上产业园区实行灵活的审批、用人、分配政策，推动园区提速发展。加快洞庭湖电子商务新城、健康医药产业园、现代装备产业园等示范基地建设。突出抓好“135”工程，建成标准化厂房259万平方米，引进创新创业企业250家以上，经开区、城陵矶新港区引进投资过20亿元项目1个以上，其他园区引进投资过5亿元项目2个以上。支持企业孵化、技术研发、质量检测等平台建设，新增著名商标20个、名牌产品12个。进一步提升港区运营效益。依托“一区一港四口岸”，大力引进国际国内500强企业、央省企业、大型企业集团和进口加工类、贸易服务类产业链项目，推动临港产业集聚发展。强化通关转关服务，加快建设江海轮船队，提高通关效率与航线运力。在更大范围内组织货源、拓展市场，延伸港口经济腹地。进一步提升景区人气指数。突出抓好岳州古城、洞庭湖博览园、环南湖“13118”综合工程、屈子文化产业园、洋沙湖国际旅游度假区、张谷英古建筑群等项目建设，丰富景区内涵。创新景区运营机制，推进景区所有权和经营权分离，加快君山岛景区（芦苇荡）和野生荷花世界景区整体开发。开展“热游岳阳”活动，加大创意及营销策划力度，重点推介3—4条精品旅游线路，运用市场手段办好特色旅游节会。进一步提升县区综合实力。牢固树立“强县才能强市”理念。按照“一核三极两区多点”空间布局，坚持宜农则农、宜工则工、宜商则商、宜游则游，走错位竞争、特色发展之路，力争3—5个县市区综合实力进入全省20强。突出县域经济“六个一”工作重点，着力解决“指挥棒”过多的问题，支持各县市区依托区位优势、资源禀赋和产业基础，培育壮大主导产业。继续开展“学长沙、赶宜昌、超九江”活动，力争2个以上县市区率先实现对标赶超，进一步昂起县域经济“龙头”，挺起市域经济“脊梁”！

三要坚决落实一批改革任务。改革是发展的动力，只有进行时，没有完成时，再难也要向前推进，特别是要从供给和需求两端发力，让我市改革取得看得见、摸得着的成效。经济体制改革：深化投融资体制改革，整合融资平台，设立城市建设发展基金，稳妥推进农商行改革。大力引进股份制商业银行和村镇银行，促进保险证券业和地方小微金融稳健发展。推进财政收入管理、预算管理、体制管理、资金管理改革，逐步建立现代财政制度。整合建立统一的公共资源交易平台。加强政府性债务管理，防范债务风险。推进住房制度改革。做好第三次全国农业普查。行政体制改革：完成公车改革和行政村调整。建立不动产统一登记制度。稳步推进事业单位分类改革、司法体制与行政执法体制改革。严格执行权力清单、责任清单、负面清单制，加快转变政府职能。社会体制改革：启动“县管校用”教师人事制度、校长职级制度改革，实行区域内校长、教师交流制度，促进教育公平。加快公立医院改革，推进基本药物制度全覆盖，落实社会办医优惠政策，探索建立分级诊疗制度。深化城市管理体制改革。推进信访制度改革，依法依规依程序处理信访问题。农村体制改革：探索推进农村产权制度改革试点，深化农村土地确权登记颁证和土地承包经营权抵押贷款等改革，依法推进农民住房财产权抵押、担保、转让，增加农民财产性收入。

四要努力破解一批发展难题。当前，我们面临的发展难题还很多，这些问题不能回避、不能遮掩，必须坚持问题导向，一个一个地具体研究、具体解决。强化要素保障。促进金融与财政、产业政策融合，加强对重大项目、中小微企业、高新产业、县域经济发展的金融支持，新增贷款100亿元以上。发展多层次资本市场，积极培育上市后备资源，构建多元融资体系。深入开展禁拆治违行动，以铁的决心、铁的措施、铁的纪律继续整治违法建设。加大土地依法征收力度，严格清查闲置土地，盘活存量土地。实施“巴陵人才工程”，促成更多中高端人才进驻岳阳，共同为改革想招、为发展出力！强化环境保障。全面落实创新创业和惠企政策，继续实施企业“宁静日”、“缴费清单”、“围墙法则”等制度，严厉整治破坏发展环境的行为。始终把人民生命安全放在首位，强化安全生产底线思维和红线意识，严格落实安全生产党政同责、一岗双责、失职追责，扎实开展“安全生产监管执法年”活动，加强社会治安综合治理和信访维稳工作，为发展提供和谐稳定的社会环境。

五要扎实办好一批民生实事。悠悠万事，民生为大。我们要始终坚持促发展与惠民生并重，认真抓好普惠性、基础性、兜底性民生建设，织牢织密民生保障网。强力推进精准扶贫，制定完善水、电、路等10个重点攻坚规划，贫困个体技能培训和就业等5个专项攻坚规划，精准施策、靶向施治，确保脱贫11.6万人。统筹推进农村危（旧）房改造与规范农村村民建房，加快2个省级示范点、10个市级示范点建设，完成4万户农村危（旧）房改造任务。突出抓好就业创业工作，新增城镇就业5.2万人、农村劳动力转移就业3.5万人、失业人员再就业2.7万人。加快中心城区中小学校“六建六扩”步伐，努力解决“大班额”问题，完成市十四中置换搬迁，启动市十六中建设，新建村小和教学点合格学校72所、农村普惠性幼儿园68所。加快市中心医院、市妇女儿童医院、市中医院骨伤大楼建设，救治救助贫困重性精神疾病患者800人，新增养老服务床位5000张。建成市广电中心，加快推进岳阳文庙修缮工程，成功创建国家公共文化服务体系示范区。我们要通过办好一件件、一桩桩民生实事，坚决托住民生底线，让广大群众幸福感、获得感更加强烈浓厚！

各位代表，未来五年，岳阳的发展任务特别繁重、责任十分重大，要做好政府各项工作，加强政府自身建设至关重要。我们将努力做到：依法用权“不任性”。每一条法律法规都是权力的“防腐剂”，是政府工作人员用权行事的“硬杠杠”。我们将始终把法治作为政府工作第一准则，带头尊法学法守法用法，自觉接受人大、政协和全社会监督。严格依法科学民主决策，严格实施“两清单两目录”，严格做到公正文明执法，为行政权力划定法治轨道，把权力关进制度“笼子”，决不允许政府工作人员目无法纪、践踏法纪。服务群众“零距离”。为人民服务是政府的天职。我们将始终把服务群众作为第一职责，

推动更多公共资源向民生聚焦、更多公共平台向基层倾斜、更多公共服务事项下放社区办理，拉近政府与老百姓的距离，让广大群众享受到更便捷、更贴心、更温暖的服务。敢于担当“重落实”。政府工作是老老实实的工作，来不得半点虚假与“花招”。我们将始终把求真务实作为第一要求，力戒形式主义、“假大空”和表面文章，凡重大决策一以贯之，坚决落地落细，决不动摇折腾；凡重大项目一抓到底，严格督促督查，决不半途而废；凡重大任务一鼓作气，全程问责问效，决不无果而终。清廉从政“守底线”。政府的每一名工作人员不论职务高低，都是人民的公仆，必须清醒认识到权力是为人民服务的工具，决不能利用手中权力谋私利、徇私情。我们将始终把廉洁从政作为第一形象，严格落实党风廉政建设“一岗双责”，严格践行“八项规定”和“三严三实”，严格强化重点领域、关键环节监管，坚决反对“五种陋习”，旗帜鲜明反腐败、反“四风”，持之以恒转作风、正行风，努力建设人民满意政府。

各位代表，岳阳发展已经站上了一个新的起点。面对“十三五”跨越发展的新任务、人民群众的新期待，让我们在中共岳阳市委的坚强领导下，协力同心、攻坚克难、开拓奋进，为全面建成小康社会、加快建设“一极三宜”江湖名城而努力奋斗！
